# Comprehensive Comparison of Similarity Evaluation and Discovery of Weak Spectral Variations of Near-Infrared Spectroscopy for Tobacco Formulation Replacement

**DOI:** 10.1155/ianc/7998333

**Published:** 2025-11-30

**Authors:** Yipeng Zhang, Hui Jiang, Jun Ling, Liliang Wen, Keliang Yan, Aiming Chen, Zhongda Zeng, Miaomiao Wang, Qianxu Yang

**Affiliations:** ^1^R & D Center, China Tobacco Yunnan Industrial Co. Ltd., Kunming 650202, Yunnan, China; ^2^Department of R & D, Dalian Chem Data Solution Information Technology Co. Ltd., Dalian 116023, Liaoning, China; ^3^College of Environmental and Chemical Engineering, Dalian University, Dalian 116622, Liaoning, China; ^4^Xinjiang Science & Technology Resource Sharing Service Center, Xinjiang Key Laboratory of Featured Functional Food Nutrition and Safety Testing, Kexue North Road 374, Urumqi 830011, Xinjiang Uygur, China

**Keywords:** data preprocessing, near-infrared spectroscopy, similarity evaluation, tobacco formulation replacement, weak spectral variations

## Abstract

Near-infrared (NIR) spectroscopy data encounter challenges in data processing such as peak overlapping, information redundancy, and background or noise, which complicate the evaluation of weak differences among similar samples. Therefore, accurately identifying these differences and assessing similarities are essential in practical applications for sample classification and further replacement of raw materials in the product formulation. In this work, 32 data preprocessing strategies of NIR data were systematically combined for comprehensive comparison, and 11 methods for similarity analysis were evaluated to attain optimal performance. Using the rationality of similarity evaluation as the assessment criterion, the combination of NIR data pretreatment methods of “standard normal variate (SNV) + first-order derivative by Savitzky–Golay (1D/SG) + maximum–minimum scaling (MMS) + spectral similarity by combinatorial strategy (SS/CS)” is ultimately preferred as the most effective combination for similarity evaluation. It uses SNV transformation, 1D/SG, MMS, and scattering correction to eliminate the scattering effect, enhance the signal-to-noise ratio (SNR) of the distinction of overlapping peaks, and improve data comparability. After this, the widely used methods for similarity evaluation were employed for comprehensive analysis and comparison of the rationality, such as Euclidean distance, correlation coefficient, and divergence information. The evaluation strategy proposed in this work can effectively distinguish the difference among the tobacco samples existing in 10 different categories. The similarity among typical samples in the same class is above 0.9, while the values in different classes are below 0.7. In real applications for method validation, recognition precision of tobacco samples with blending of interfering mixtures reaches 5%, which is conducted using complex tobacco materials for formulation replacement and optimization. The satisfactory results introduce robust and CS that outperforms traditional single-method approaches to resolve weak spectral differences through real-world tobacco formulation replacement applications. It can be widely used in the areas related to NIR for similarity evaluation, such as pharmaceuticals, food quality control, and environmental monitoring.

## 1. Introduction

Near-infrared (NIR) spectroscopy is a spectroscopic technique that analyzes the electromagnetic waves in the region between visible and mid-infrared light [1, 2]. It is primarily characterized by the absorption caused by the nonharmonic vibrations of molecules [3]. This absorption occurs during molecular transitions from the ground state to higher energy levels, which primarily reflects the vibrational frequencies and amplitudes of hydrogen-containing groups, such as C-H, N-H, and O-H. Different groups or the same group existing in different chemical environments generate significant differences in both dimensions of absorption wavelength and intensity of NIR. Thus, it enables the reflection of structural and compositional information of substances or samples with informative chemical structures [4, 5]. Variations in the quantity and structural forms of groups containing hydrogen and other elements in the compounds result in the corresponding information in NIR transmission or reflection spectra. Over 80%–90% of crucial feature information and chemical structure of organic components can be expressed in the NIR region [6]. This technology offers multiple advantages, including rapid detection, cost-effectiveness, and nondestructive analysis. It has found extensive applications and development in various fields, such as food [7], pharmaceuticals [8], and agricultural products [9].

The absorption wavelengths and intensities in NIR may vary significantly among different substances. Using suitable methods to analyze the NIR of analytical samples, it is possible to identify the relationships between various chemical components in the samples, interpret sample features, and distinguish similarities among samples [10, 11]. However, interference information, such as background and noise, unavoidably exists in the experimental measurement [12], which directly impacts the results of NIR analysis and can even lead to incorrect conclusions. Therefore, it is necessary to perform data preprocessing on NIR to eliminate the influence of irrelevant contribution while retaining the spectral response of targeted compounds, which helps to interpret sample characteristics [13] robustly.

However, NIR data simultaneously include the characteristics of many compounds, and it needs help to directly construct models for qualitative or quantitative analysis, such as peak overlapping, information redundancy, nonlinearity, and coexistence of complex interference [12, 14]. In particular, the hybrid information of multiple compounds with high similarity of NIR measured from samples with high correlation makes it difficult to discover the weak difference among data for real applications. For example, replacement or optimization of partial raw materials in formulation design or product manufacturing has great importance and challenge to attain quality inspection for the samples with similar changes in time and space dimensions [15, 16]. This could result in subtle spectral signal variations within NIR datasets and then complicate sample differentiation and pattern recognition during comparative analysis via similarity/difference metrics and quantitative modeling [17, 18]. These indistinguishable spectral signal differences might also be obscured in the presence of similar compounds and background models. Therefore, appropriate preprocessing methods and similarity evaluation strategies are required to accurately identify these weak spectral signals and then discern minor variations among samples for applications [19].

Statistical and chemometric analysis, computer technology, and other techniques have provided numerous data preprocessing methods and NIR similarity evaluation strategies. For instance, the widely used methods for NIR preprocessing include standardization for constructing models of low-concentration components [20], derivative analysis for eliminating baseline and background interference [21], standard normal variate (SNV) transformation [14], multiple scattering correction (MSC) to remove NIR diffuse reflectance effects [22], wavelet transform for spectral signal smoothing and noise filtering [23], and many other methods for data operations. Various similarity methods are used to evaluate the similarity between NIR data of related samples to ensure product quality consistency and reliability, detect anomalies in the production process, identify and optimize sample components and formulations, and classify and distinguish samples. These methods have great significance to maintain product quality, improve production efficiency, and optimize formulations in food, pharmaceuticals, and agriculture areas. The standard similarity evaluation methods for NIR analysis involve calculating metrics, such as Pearson's correlation coefficients [24], Euclidean distance, cosine angle, Manhattan distance [17], and relative entropy [5], between NIR data to evaluate their similarity and difference quantitatively. These methods have been widely applied for the assessment of NIR data. In certain application areas, the difference between samples may primarily manifest as weak spectral variations related to low-concentration substances, which may be incorrectly treated as interference and not be effectively preserved, which may lead to adequate employment during the similarity evaluation process. Thus, reasonable methods for simultaneous NIR data preprocessing and similarity evaluation are required to discover information hidden in NIR data effectively. It should simultaneously consider the compatibility between preprocessing and similarity evaluation, and further aim to extract the weak signal. Then, it can further prompt the application of NIR technology in many fields, such as quality inspection and formulation design with the help of rich NIR signals for similarity evaluation.

In this work, it systematically investigated various data preprocessing and similarity evaluation methods and their compatibility. This helps to enhance the ability to discover and assess weak but significant NIR differences among samples. It comprehensively analyzes 32 preprocessing methods and 11 similarity evaluation methods. Using some specific cases as examples for NIR data analysis and similarity evaluation, the proposed strategy was demonstrated and further validated to discover weak differences. It generates valuable results in NIR areas for wide applications, as introduced above.

## 2. Theory

**Figure 1:**
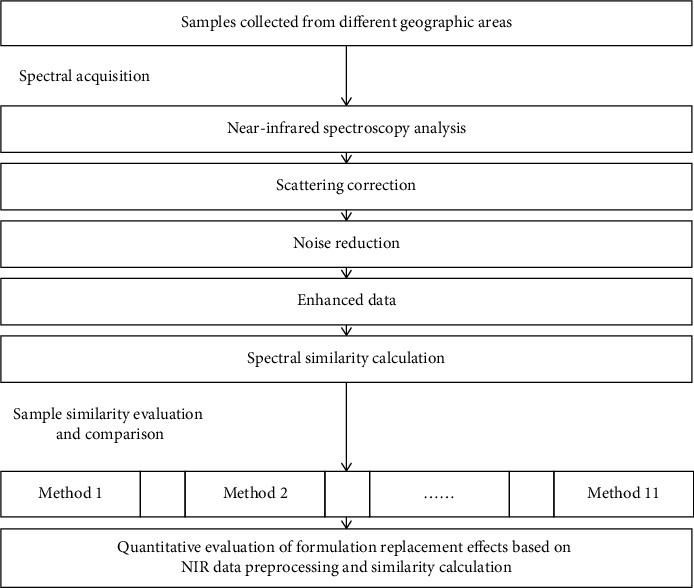
Flowchart of this work for simultaneous data pretreatment and similarity evaluation to discover weak sample differences in NIR data.

Data preprocessing is a crucial step in NIR analysis, which has significant influence to the results. It is essential to effectively remove or reduce the interference of background and noise, improve data quality, and enhance the NIR signal of target compounds, which is essential for subsequent qualitative and quantitative analyses, such as similarity evaluation and quantitative model development. As shown in Figure 1, the basic workflow for quantitative similarity evaluation on the basis of NIR data preprocessing is outlined for reference. In this work, it employs the steps and methods illustrated in this figure to extract weak NIR information and then perform similarity evaluation for precise discovery of weak differences among sample.

As shown in Figure 1, a series of data preprocessing steps, including scattering correction, noise reduction, and data enhancement, are required for NIR data handling to eliminate the influence of buried interfering information. These steps are designed to reveal weak differences among spectra, which enables similarity analysis between spectra and evaluation of characteristics of sample variation.  Table 1 introduces the standard methods and their primary functions for NIR data pretreatment.

It is not difficult to understand that different similarity methods for data evaluation focus on or emphasize certain features within the data structure. Therefore, they often yield not entirely consistent analytical performance or results, and also have a degree of adaptability to data with different structures or characteristics. Thus, it highlights the importance of selecting appropriate data preprocessing methods and similarity comparison metrics. In this work, the similarity evaluation methods are shown in Table 2.

As given in Table 2, the similarity evaluation of NIR data employs various methods for complete comparison, each with unique principles and mathematical foundations. The Pearson correlation coefficient (M1) measures spectral similarity based on the correlation between two spectra, which takes into account spectral anomalies and noise. This method effectively identifies linear relationships but can be sensitive to outliers. In contrast, the Euclidean distance (M2) evaluates similarity based on the amplitude differences between spectra, which makes it insensitive to shape differences but potentially less effective in distinguishing spectra with similar amplitudes but different shapes. The spectral angle mapper (SAM, M3) assesses similarity based on the spatial angle between spectral vectors and prioritizes spectral shape over amplitude. It makes it suitable for detecting shape similarities even when amplitudes have difference. Comparatively, the Manhattan distance (M4) calculates the absolute differences between spectra and provides a straightforward measure of similarity but lacks sensitivity to spectral shape.

The index of spectral information divergence (SID, M5) uses relative entropy (Kullback–Leibler divergence) to measure similarity, offering a probabilistic approach that can capture more nuanced spectral differences but may require careful handling of probability distributions. The combined methods, such as ED/COD (M6) and SS/CS (M7), integrate Euclidean distance and correlation coefficients to leverage both strengths of the metrics, which enhances robustness and sensitivity in similarity evaluation. Other methods, such as Hsim (M8), Close (M9), Gsim (M10), and Esim (M11), address the limitations of traditional distance measures, particularly in high-dimensional spaces. For example, Hsim introduces a harmonic mean approach to balance differences, while Close employs exponential transformations to mitigate the impact of high dimensionality. Gsim and Esim further refine these concepts and incorporate weights and enhance accuracy through more complex mathematical formulas.

As introduced above, each similarity method offers distinct advantages and is supported by specific mathematical theories. Pearson's and Euclidean methods are straightforward and widely applicable, whereas SAM and Manhattan provide more specialized measures. Combining multiple metrics, SID, and combinatorial methods, such as ED/COD and SS/CS, offers robust, nuanced evaluations. Advanced methods, such as Hsim, Close, Gsim, and Esim, enhance traditional approaches and address the challenges of high-dimensional NIR data. The selection of appropriate methods depends on the requirements of NIR data analysis, including the importance of amplitude versus shape similarity and handling of high-dimensional data. Of course, this work aims to make an essential comparison.

Using the NIR preprocessing methods and similarity evaluation strategies introduced above, it focuses on the quantitative evaluation of NIR with weak differences in this work. For example, it employs replacing raw tobacco materials with low blending ratios in formulation design. It aims to provide objective and scientific support for various application scenarios based on NIR technology, such as quality inspection and material replacement in formulations.

**Figure 2:**
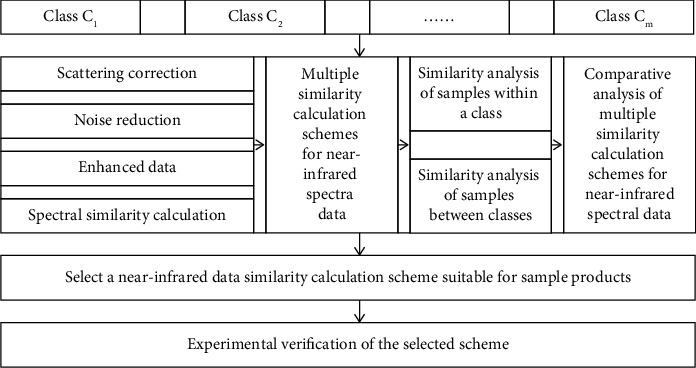
The overall scheme for NIR similarity evaluation.

Figure 2 illustrates the overall research scheme of this work. The data preprocessing methods for NIR, such as scattering correction, noise reduction, and data enhancement, are shown in Table 1. Based on this foundation, there are a total of (1 + 1) × (1 + 3) × (1 + 3) = 32 data preprocessing schemes, where the first number “1” represents not performing any algorithm operation given in Table 1. The second numbers 1, 3, and three in each pair correspond to the number of methods for the three kinds of strategies for data processing. The total number is 7, as shown in Table 1. After this, different similarity evaluation methods of the processed NIR data are applied, as shown in Table 2. This results in a comprehensive and systematic comparative analysis of 32 × 11 = 352 times of simultaneous NIR processing and similarity evaluation. These analytical operations are then used to select the most suitable approaches for similarity analysis, which allows for the replacement of low blending ratios of materials in formulations. This provides the basis for the evaluation of weak differences in NIR data.

## 3. Experimental Section

### 3.1. Spectral Acquisition

In this work, 51 raw tobacco samples were collected from different geographic regions of Yunnan Province of China with different species and tobacco parts. These samples can be categorized into 10 groups, which serve as the basis for subsequent similarity evaluation and further applications. The information on specific sample classification is detailed in Table 3.

The NIR spectrometer (Antaris II, Thermo Fisher Scientific) was applied for NIR measurement under the same experimental conditions to all the 51 samples. The specific sample preparation methods and experimental conditions are detailed as follows:

For sample preparation, a multistep process was implemented. First, for moisture adjustment, samples were continuously dried in a 40°C oven. The goal of this drying process was to adjust the sample moisture content to a range of 6%–8%. This precise moisture level is crucial as it can affect the subsequent NIR analysis, as moisture content variations may lead to inconsistent spectral responses. Subsequently, the samples underwent grinding. A grinder was used to crush the samples, and then, the ground samples were passed through a 0.250-mm (60-mesh) sieve. Passing through the sieve ensures that the sample particles are of a uniform size, which is important for reproducible NIR measurements. Uniform particle size helps in achieving consistent light scattering and absorption during the NIR analysis. Finally, sample mixing was carried out. A blender was employed to thoroughly mix the powder. This step is aimed at ensuring uniform consistency of the samples for measurement. By complete mixing, any potential inhomogeneities in the sample are eliminated, which is essential for obtaining reliable and reproducible NIR spectra.

The experimental conditions for NIR analysis were strictly controlled. The environmental temperature was maintained within a range of 25°C–28°C. The temperature stability is vital in NIR analysis because temperature fluctuations can influence molecular vibrations and then spectral features. In addition to temperature, the environmental relative humidity was set to 60%–65%. Relative humidity can impact the moisture content of samples even after the initial moisture adjustment, and may also affect the performance of optical components in the NIR instrument.

Regarding the instrumental parameters for NIR measurement, the scanning range was set from 10,000 cm^−1^ to 4000 cm^−1^. This range covers a broad spectrum of NIR wavelengths, which is typical for analyzing molecular vibrations and overtones relevant to various chemical components in the samples. The resolution was configured to 8 cm^−1^. The resolution determines the ability of the instrument to distinguish between closely spaced spectral features. An 8 cm^−1^ resolution is a commonly used setting in NIR analysis, which balances between spectral details and SNR requirements. The number of scans was set to 64. A higher number of scans, such as 64, help in improving the SNR of NIR spectra. By averaging the signals from multiple scans, random noise is reduced to result in more accurate and reproducible spectral data.

To the samples introduced in Table 3, the tobacco leaves within the same class share the same place of origin, variety, and part. As a result, the samples with the same class should exhibit high similarity of NIR data, and vice versa.

### 3.2. Data Processing

After collecting the NIR of all experimental samples, the 352 combinations of spectral preprocessing and similarity evaluation methods introduced above were designed and applied for data analysis. The similarity indices were calculated within each sample class and between different classes. To ensure comparability of the results, the similarity measurement generated outside the range of [0, 1] was normalized to the 11 methods.

To a specific method for similarity evaluation given in Table 2, the similarity values of the samples within and between classes are, respectively, defined in equations (1) and (2). The sample information within or between classes is illustrated in Table 3.

(1) $$\begin{array}{c} \text{Within Clas}s_{i}=\frac{\sum_{k=1}^{10}\sum_{p,q\in \text{Clas}s_{k},p\neq q}\text{Si}m_{i}\left(P,Q\right)}{n}, \end{array}$$

(2) $$\begin{array}{c} \text{Between Clas}s_{i}=\frac{\sum_{k,l\in \text{Class},k\neq l}\sum_{p\in \text{clas}s_{k},q\in \text{Clas}s_{l}}\text{Si}m_{i}\left(P,Q\right)}{m}, \end{array}$$

In equations (1) and (2), Sim_*i*_(*P*, *Q*) represents the similarity results between the original or preprocessed NIR data of two samples, *P* and *Q*, respectively. The total number of similarity values of the exhaustive combination of 11 similarity methods and 32 NIR data is 352 (11 × 32), as introduced above. In equation (1), “10” indicates the number of sample classes, as shown in Table 3. The letters *P* and *Q* represent the NIR data of two samples existing in the same class, and the letter *n* means the number for similarity evaluation within the class to each method. Equation (2) calculates the similarity of all sample pairs existing in different classes *k* and *l*. Correspondingly, the letter *m* means the number for similarity calculation between classes to each method, and the two letters *P* and *Q* represent the NIR data of two samples existing in different classes, *k* and *l*. It has no difficulty to understand that the results of “Within Class_*i*_” and “Between Class_*i*_” are used to quantify Sim_*i*_'s similarity evaluation for the NIR data of similar samples existing in the same class or differential samples between different classes. As mentioned above, the “Class” in both equations (1) and (2) represents all the 10 sample classes, as given in Table 3.

## 4. Results and Discussion

Based on equations (1) and (2), a total of 352 combinations of spectral preprocessing and similarity evaluation methods were wholly used to calculate the similarity results within and between classes of samples, respectively.

**Figure 3:**
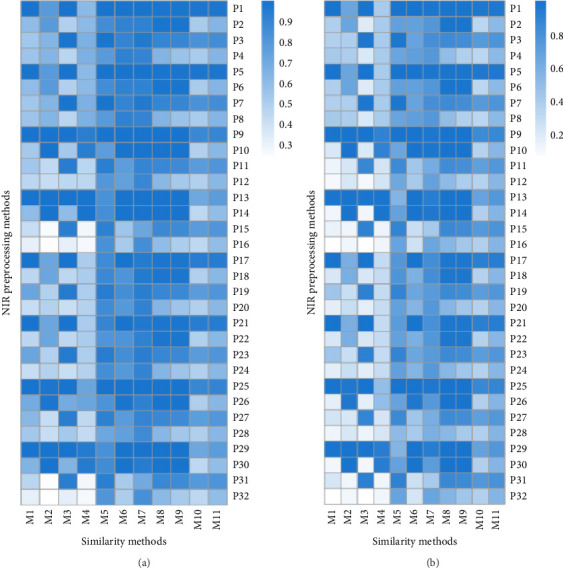
Heatmap showing the similarity results obtained from (a) within class and (b) between classes. The values of mean and standard deviation of each method for similarity evaluation were 0.64 ± 0.23, 0.66 ± 0.26, 0.73 ± 0.26, 0.6 ± 0.22, 0.88 ± 0.08, 0.86 ± 0.16, 0.93 ± 0.07, 0.88 ± 0.15, 0.85 ± 0.19, 0.66 ± 0.19, 0.74 ± 0.13, and 0.43 ± 0.35, 0.53 ± 0.32, 0.56 ± 0.41, 0.43 ± 0.27, 0.77 ± 0.14, 0.7 ± 0.27, 0.77 ± 0.15, 0.84 ± 0.19, 0.81 ± 0.24, 0.58 ± 0.23, and 0.69 ± 0.16 to the results shown in (a) and (b), respectively.

Figures 3(a) and 3(b), respectively, show the similarity evaluation results for the samples within and between classes using different methods. The specific values of mean and standard deviation (SD) of each method were introduced in the figure captions. In the two heatmaps, the abscissa and ordinate axes represent the 11 similarity methods for spectral evaluation, as shown in Table 2, and the 32 spectral preprocessing combinations, respectively. The color intensity of each cell indicates the similarity value for the corresponding NIR data under a specific similarity method with darker colors representing higher similarity values. In the first heatmap, most data preprocessing methods exhibit higher similarity results across most similarity methods (darker colors). Specifically, methods M5, M6, and M7 demonstrate consistently higher similarity results across most preprocessing methods, which indicates higher effectiveness in intraclass analysis. Additionally, preprocessing methods P1, P3, P5, P6, P10, and P18 yield better results under most similarity methods and indicate higher intraclass similarity. The second heatmap displays the interclass similarity results for different data preprocessing methods with a similar illustration to the first heatmap. Compared to the heatmap in Figure 3(a), the overall similarity results in the second heatmap are generally lower (lighter colors). Except for M8 and M9, other methods exhibit relatively higher similarity results across most preprocessing methods. This suggests their effectiveness in interclass analysis. Moreover, the preprocessing methods of P4, P8, P12, P16, P20, P24, P28, and P32 show lower similarity results under most similarity methods. It indicates relatively poor interclass correlation.

A comparative analysis of the intraclass and interclass results reveals that the similarity values of intraclass are generally higher. This indicates a higher degree of feature similarity within the same class. In contrast, interclass similarity values are generally lower and indicate lower feature similarity between different classes, as expected. Regarding the effectiveness of similarity methods, M5 and M6 consistently show relatively satisfying results of similarity values in both intraclass and interclass analyses. The analysis of these two heatmaps reveals significant impacts of different data preprocessing strategies and similarity methods on the evaluation results of NIR data. These findings underscore the importance of selecting appropriate data preprocessing techniques and similarity methods to enhance the effectiveness of analysis in NIR data.

In the comparative analysis of intraclass results shown in Figure 3(a), methods M1 to M4 and M10 to M11 are noted to have relatively higher SD in similarity values among different algorithms for data preprocessing, which indicates more significant variability and lower consistency compared to other methods.  Figure 3(b) also exhibits a similar trend in the SD of similarity results. In the analysis, the wider confidence intervals associated with interclass similarity values indicate more significant uncertainty in the estimation of similarity parameters across different classes. This underscores the challenges in consistently measuring and interpreting similarity between diverse sample groups in NIR data.

These statistical insights complement the visual representations provided by Figures 3(a) and 3(b), and offer a robust framework to assess the reliability and significance of intraclass and interclass similarity analyses. By emphasizing these statistical aspects, the study provides a comprehensive understanding of how different data preprocessing techniques and similarity methods influence the consistency and variability of NIR data in quantitative evaluations of sameness and difference. In equation (3), the integrated ability of methods for similarity evaluation of NIR data was quantitatively defined to differentiate the samples within and between classes. A higher value of this index indicates that it performs better in extracting the difference of spectral information among samples and can be used for more effective similarity assessment.

(3) $$\begin{array}{c} \text{Evaluation Index}=\frac{\left(\text{Within Class}+\left(1-\text{Between Class}\right)\right)}{2}. \end{array}$$

In NIR data similarity analysis, the hybrid index is a crucial metric for assessing the correlation of samples within or between classes. This index is formulated to account for both the intraclass similarity and interclass dissimilarity. The “Within Class” component measures how closely related the NIR data are within the same class, and higher values indicate greater compactness and homogeneity within a class. It is desirable as this suggests that the similarity measurement accurately correlates similar NIR spectra together. Conversely, the “Between Class” component assesses the similarity between NIR data from different classes. By incorporating “1- Between Class” into the index, the formulation ensures that lower similarity between different classes contributes positively to the overall evaluation and reflects better class separation. An increasing in the “Within Class” value or a decrease in the “Between Class” value will both lead to a higher value of the “Evaluation Index,” which signifies an improved performance for similarity measurement. The balanced consideration of both within-class and between-class metrics makes the index an effective tool for evaluating the quality of NIR data similarity analysis. This ensures that the classes are compact and well-separated.

Through the approach proposed above, the similarity assessment schemes were selected that could effectively distinguish the similar samples within the same class (within class ≥ 0.9) and the differential samples between different classes (between class < 0.7), as shown in Table 4.

**Figure 4:**
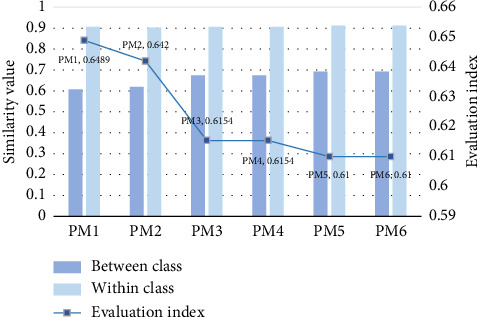
Similarity value for different evaluation methods. The horizontal coordinates represent different methods, and the left and right vertical coordinates represent similar results and integrated evaluation indexes.

As shown in Figure 4, the similarity value for the “SNV+1D/SG + MMS + SS/CS” (PM1) scheme generates the highest value of 0.65. This indicates that it performs better results in the process for comprehensive similarity assessment of sample. Thus, it was selected to analyze NIR data preprocessing and similarity comparison.

The spectral preprocessing of “SNV+1D/SG + MMS + SS/CS” and similarity evaluation method involves the following steps:

Step 1: In the process of the preprocessing step of NIR data, it was handled as follows:

1. SNV transformation eliminates the interference of scattering effects during data collection.
2. 1D/SG removes the influence caused by environmental background and electrical interference and further enhances the measurement signal for analysis.
3. The MMS method enhances the overall contribution of weak spectral information and effectively removes the interference of stray light, environmental background, and electrical noise.

Step 2: In the process of similarity evaluation step of the processed NIR data, it was handled as follows:

1. The method of combinatorial strategy of Euclidean distance, Pearson's correlation coefficient, and information divergence is used for a comprehensive assessment of spectral similarity (SS/CS).
2. The SS/CS method ensures a more holistic evaluation of spectral similarity by considering multiple similarity metrics.
3. It avoids the potential bias of a single evaluation metric, which may be introduced by neglecting or overemphasizing the weak spectral difference between samples.

As a result, the combination of “SNV + 1D/SG + MMS + SS/CS” was chosen as the method to explore and evaluate the weak spectral signal in real applications, such as quality inspection of samples in time and space dimensions or sample replacement or optimization in industry production, as introduced above.

In this study, the weak difference among different NIR data was explored, and the similarity evaluation methods of NIR with high correlation were investigated. It has essential importance in many industrial areas, such as material replacement during the process of product design or manufacturing. Using the replacement of raw materials in the formulation design of cigarette products as an example, the leaf group formulations typically consist of dozens of different tobacco leaf materials, in which each type of tobacco leaf material only has a small proportion in the cigarette formulation (usually between 2.5% and 10%). As the quantity of tobacco leaf materials and their corresponding quality have been permanently changed, replacement of some of the tobacco leaf materials in the formulation may be necessary without affecting the integrated stability of cigarette product quality. Therefore, accurately identifying the weak difference of NIR information before and after replacement of small proportions of tobacco leaf materials in the formulation and then further evaluating the similarity of the two formulations has excellent significance to attain the goal of maintaining cigarette quality. Here, an example of the validation process and results for replacing tobacco leaf materials in cigarette formulations was given to deliver the strategy introduced above.

As shown in Table 5, the formulation replacement application including 6 cases from E1 to E6 was first created, and the same NIR data acquisition method, as mentioned above, was used to acquire the data for raw material replacement.

**Figure 5:**
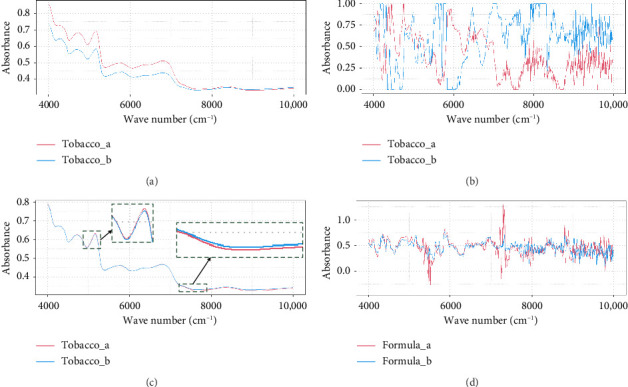
The original NIR data for formulation replacement and the process for data pretreatment. (a) The original NIR spectra of tobacco_a and tobacco_b; (b) the preprocessed NIR spectra of tobacco_a and tobacco_b by using the “SNV + 1D/SG + MMS” method; (c) the original NIR spectra of formula_a and formula_b; (d) the preprocessed NIR spectra of formula_a and formula_b using the “SNV + 1D/SG + MMS” method.

In the case of experiment E5, as shown in Table 5, a small proportion replacement experiment with differential tobacco leaves was conducted as an example. In this experiment, the raw materials called tobacco_a constitute 5% of the formula_a, which was replaced in equal proportion with tobacco_b to create a new formula, namely formula_b. The comparison of NIR data before and after data pretreatment using the “SNV+1D/SG + MMS + SS/CS” method is depicted in Figure 5.

Figure 5(a) and 5(b) show the original and preprocessed NIR spectra of samples for formulation replacement. As shown in Figure 5(c), there is only a very weak difference in the original NIR of the formulations between 4900 nm and 5100 nm and 7200 nm–8000 nm after extremely low replacement of ingredients, namely 5% in this case. However, the original weak NIR difference has been amplified. It can be ideally used to distinguish the difference between the formulations before and after replacement of raw tobacco materials by using the preprocessing strategy of “SNV+1D/SG + MMS,” as given in Figure 5(d). This indicates that the “SNV + 1D/SG + MMS” preprocessing method can retain the weak NIR information.

From the experimental scenarios E1 and E2, it can be observed that while using similar materials (with similarity attaining 0.96) for low-proportion replacement (5% and 7.5%), the similarity between the formulations before and after the replacement remains very high, which correspond to the similarity of 0.998 and 0.997, respectively. The experimental scenarios E3 and E4 show that when using similar materials with a similarity of 0.979 for high proportion replacement, namely 25% and 27.5%, the similarity between the formulations before and after replacement remains relatively high. However, there is a noticeable decrease for the lower proportion replacements, namely 0.984 and 0.985, respectively. Finally, it can be observed that while using differential materials with similarity lower than 0.3 for low-proportion replacement, namely 5.0% and 7.5% of cigarette formulation in the experimental scenarios E5 and E6, there is a significant decrease in the similarity between the formulations before and after replacement, namely 0.944 and 0.940, respectively.

As introduced above, the analytical results indicate that the “SNV+1D/SG + MMS + SS/CS” processing and further similarity evaluation can accurately reflect the similarity of the replaced tobacco leaves and their replacement ratios to maintain raw materials formulation in cigarette. This suggests that the proposed strategy can effectively and precisely identify weak signal differences in NIR data of tobacco leaf samples. It utilizes the weak differential information in NIR to identify the replacement of low-proportion materials.

As introduced above, the discovery and employment of weak signal differences in NIR data are of significant importance in many real scenarios. Accurate and reliable similarity and difference evaluations enable precise identification of raw material replacement in formulation maintenance, which facilitates accurate assessment of the effectiveness of product maintenance. This is conducive to advancing the “digital design” process of formulation management and further design of new products.

## 5. Conclusion

The inherent complexity of NIR data poses significant challenges for accurate similarity evaluation and difference detection, including overlapping signals and subtle spectral variations. At the same time, the growing demand for high-throughput, nondestructive analysis in fields, such as quality control, formulation design, and environmental monitoring, necessitates more robust strategies to distinguish weak differences among similar samples. In this study, a comprehensive evaluation of 32 data preprocessing strategies and 11 similarity evaluation methods was conducted. Based on this systematic investigation, an optimized strategy integrating SNV + 1D/SG + MMS + SS/CS was proposed. This combination effectively enhances weak signal components and improves the reliability of similarity measurements in NIR data. The proposed method demonstrated excellent performance in a practical and critical application, namely, replacement of raw tobacco materials in cigarette formulation design. The similarity scores within the same formulation class exceeded 0.9, while those between distinct classes fell below 0.7, which indicates strong discriminatory power. Notably, the strategy enabled a recognition precision of 5% in adjusting cigarette blending ratios, and underscoring its capacity for detection of subtle compositional shifts. This represents a meaningful advancement over traditional empirical or trial-and-error methods, particularly in formulation optimization scenarios where precision is vital. Beyond cigarette applications, the developed framework offers broad utility. For example, it can support batch-to-batch consistency checks and early detection of raw material variation in the pharmaceutical industry.

Compared with conventional analytical techniques, such as chromatography or mass spectrometry, which offer high specificity and sensitivity, NIR spectroscopy, particularly when enhanced by the proposed preprocessing and similarity evaluation strategy, offers substantial advantages in terms of speed, cost-efficiency, nondestructive measurement, and field deployability. This makes it especially well-suited for real-time screening, high-throughput quality control, and portable on-site diagnostics. Nevertheless, the high variability of NIR signals and the influence of environmental factors, such as temperature and humidity, necessitate further refinement of preprocessing techniques and model generalization strategies. Signal overlap, despite methodological advancements, remains a constraint. Future research should aim to validate the proposed strategy across a broader array of sample types and application contexts, and integrate adaptive algorithms or machine learning models to improve robustness.

## Figures and Tables

**Table 1:** The most widely used methods for NIR data pretreatment.

No.	Name	Purpose	Method description
1	Standard normal variate (SNV) [25, 26]	Scattering correction	To eliminate the scattering effects caused by uneven sample distribution during the spectral acquisition process.

2	Data smoothing by the Savitzky–Golay (SG) method [27]	Denoising	To reduce random noise and improve the signal-to-noise ratio (SNR) of the spectra.

3	First-order derivative by the SG method (1D/SG) [28]	Signal enhancement	To remove the constant baseline and enhance the NIR signal.
4	Second-order derivative by the SG method (2D/SG) [28]		To remove the linear baseline and enhance the NIR signal.

5	Mean centering (MC) [29]	Data normalization	Correct data shift to the data center from the origin to enhance the difference between sample spectra.
6	Maximum–minimum scaling (MMS) [29]		To linearly transform the raw data to the range [0,1], which helps to detect spectral variations caused by small changes in optical path length.
7	Autoscaling (AS) [29]		To standardize NIR data for the removal of the dimensional effects.

**Table 2:** The 11 methods for similarity comparison used in this study.

No	Name	Formula	Characteristics
M1	Pearson's correlation coefficient [30]	$\text{Pearson}\left(X,Y\right)=\sum_{i=1}^{n}\left(x_{i}-\overset{¯}{x}\right)\left(y_{i}-\overset{¯}{y}\right)/\sqrt{\sum_{i=1}^{n}{\left(x_{i}-\overset{¯}{x}\right)}^{2}\sum_{i=1}^{n}{\left(y_{i}-\overset{¯}{y}\right)}^{2}}$	To evaluate spectral similarity based on correlation and consider the impact of spectral anomalies and noise on the evaluation results.

M2	Euclidean distance [31]	$\text{Euclidean}\left(X,Y\right)=\sqrt{\sum_{i=1}^{n}{\left(x_{i}-y_{i}\right)}^{2}}$	To evaluate spectral similarity based on the amplitude difference, being insensitive to spectral shape difference.

M3	SAM similarity [32]	$\text{SAM}\left(X,Y\right)=\left(\sum_{i=1}^{n}x_{i}y_{i}\right)/\sqrt{\sum_{i=1}^{n}{x_{i}}^{2}\sum_{i=1}^{n}{y_{i}}^{2}}$	To evaluate spectral similarity based on the spatial angle between spectral vectors, being sensitive to spectral shape difference but insensitive to amplitude value difference.

M4	Manhattan distance [33]	Manhattan(*X*, *Y*) = ∑_*i*=1_^*n*^|*x*_*i*_−*y*_*i*_|	To evaluate spectral similarity based on the absolute distance between spectra.

M5	SID similarity [34]	SID(*X*, *Y*) = *D*(*X*‖*Y*) + *D*(*Y*‖*X*)	To evaluate spectral similarity based on relative entropy (Kullback–Leibler divergence) between spectra.

M6	ED/COD combination [30, 31]	ED/COD(*X*, *Y*) = (1 − Pearson(*X*, *Y*)) × Euclidean(*X*, *Y*)	To evaluate spectral similarity by combining both Euclidean distance and correlation coefficient.

M7	Spectral similarity by combinatorial strategy (SS/CS) similarity [35]	SS(*X*, *Y*) = SID(*X*, *Y*) × Euclidean(*X*, *Y*) × (1 − Pearson(*X*, *Y*))	Evaluate spectral similarity by combinatorial strategy of spectral information divergence, Euclidean distance, and correlation coefficient.

M8	Hsim distance [36]	Hsim(*X*, *Y*) = (∑_*i*=1_^*n*^1/(1+|*x*_*i*_−*y*_*i*_|))/*n*	Various approaches will be used to address the issue of traditional distance measurement methods becoming ineffective in high-dimensional data space, and aim to enhance the accuracy of spectral similarity assessment.
M9	Close distance [37]	Close(*X*, *Y*) = (∑_*i*=1_^*n*^*e*^−|*x*_*i*_−*y*_*i*_⁣|^)/*n*	
M10	Gsim distance [38]	Gsim(*X*, *Y*) = ∑_*i*=1_^*n*^1 − (|*x*_*i*_−*y*_*i*_|)/(|*x*_*i*_−*y*_*i*_|+*m*_*i*_)/*n*	
M11	Esim distance [39]	Esim(*X*, *Y*) = ∑_*i*=1_^*n*^*w*_*i*_*e*^(|*x*_*i*_−*y*_*i*_|/(|*x*_*i*_ − *y*_*i*_| + |*x*_*i*_ + *y*_*i*_|)/2)^/*n*	

*Note:* In the table, *x*_*i*_ and *y*_*i*_, respectively, represent the *i*th elements of the vectors *x* and *y* obtained from NIR analysis of two samples. A symbol with a horizontal line above the letter denotes the mean value of the NIR data.

**Table 3:** Detailed information of the samples used for investigation in this work.

No.	Class no.	Geographic origin	Species	Parts	Number of samples
1	Class 1	Baoshan	K326	Middle	4
2	Class 2	Baoshan	Hongda	Upper	3
3	Class 3	Honghe	K326	Middle	6
4	Class 4	Honghe	Yun87	Upper	5
5	Class 5	Honghe	Yun87	Middle	10
6	Class 6	Kunming	Hongda	Upper	3
7	Class 7	Kunming	Hongda	Middle	8
8	Class 8	Qujing	K326	Middle	4
9	Class 9	Qujing	Yun	Middle	4
10	Class 10	Qujing	Yun	Lower	4

**Table 4:** The similarity results of samples by using different methods.

Method ID	Data preprocessing	Within class	Between class	Evaluation index
PM1	SNV + 1D/SG + MMS + SS/CS	0.91	0.61	0.65
PM2	1D/SG + MMS + SS/CS	0.90	0.62	0.64
PM3	SG + MMS + ED/COD	0.91	0.67	0.61
PM4	NN + MMS + ED/COD	0.91	0.67	0.62
PM5	NN + MC + ED/COD	0.91	0.69	0.61
PM6	SG + MC + ED/COD	0.91	0.69	0.61

*Note:* It only includes results with within class ≥ 0.9 and between class < 0.7.

**Table 5:** The scheme for formulation replacement.

ID no.	Similarity between the original and alternative materials	The proportion of substituted tobacco leaves in the formulation (%)	Similarity of the formulation before and after material replacement
E1	0.96	5	0.998
E2	0.96	7.50	0.997
E3	0.98	25	0.985
E4	0.98	27.5	0.984
E5	0.24	5	0.944
E6	0.27	7.50	0.940

*Note:* The similarity calculations for tobacco formulation during the experimental process were all conducted using the “SNV + 1D/SG + MMS + SS/CS” method.

## Data Availability

The data that support the findings of this study are available on request from the corresponding author. The data are not publicly available due to privacy or ethical restrictions.
